# Targeting Glycogen Synthase Kinase 3 Beta Regulates CD47 Expression After Myocardial Infarction in Rats via the NF-κB Signaling Pathway

**DOI:** 10.3389/fphar.2021.662726

**Published:** 2021-07-19

**Authors:** Li-Na Xu, Shu-Hui Wang, Xue-Ling Su, Sumra Komal, Hong-Kun Fan, Li Xia, Li-Rong Zhang, Sheng-Na Han

**Affiliations:** ^1^ Department of Pharmacology, School of Basic Medical Sciences, Zhengzhou University, Zhengzhou, China; ^2^ Department of Physiology, School of Basic Medical Sciences, Zhengzhou University, Zhengzhou, China; ^3^ Department of Anesthesiology in Surgery Branch, The Second Affiliated Hospital, Zhengzhou University, Zhengzhou, China

**Keywords:** glycogen synthase kinase 3 beta, integrin-associated protein, myocardial infarction, primary rat cardiomyocytes, NF-κB signaling pathway, hypoxia

## Abstract

The aim of this study was to investigate the effects of the GSK-3β/NF-κB pathway on integrin-associated protein (CD47) expression after myocardial infarction (MI) in rats. An MI Sprague Dawley rat model was established by ligating the left anterior descending coronary artery. The rats were divided into three groups: Sham, MI, and SB + MI (SB216763) groups. Immunohistochemistry was used to observe the changes in cardiac morphology. A significant reduction in the sizes of fibrotic scars was observed in the SB + MI group compared to that in the MI group. SB216763 decreased the mRNA and protein expression of CD47 and NF-κB during MI. Primary rat cardiomyocytes (RCMs) and the H9c2 cell line were used to establish *in vitro* hypoxia models. Quantitative real-time PCR and western blotting analyses were conducted to detect mRNA and protein expression levels of CD47 and NF-κB and apoptosis-related proteins, respectively. Apoptosis of hypoxic cells was assessed using flow cytometry. SB216763 reduced the protein expression of CD47 and NF-κB in RCMs and H9c2 cells under hypoxic conditions for 12 h, and alleviated hypoxia-induced apoptosis. SN50 (an NF-κB inhibitor) also decreased CD47 protein expression in RCMs and H9c2 cells under hypoxic conditions for 12 h and protected cells from apoptosis. GSK-3β upregulates CD47 expression in cardiac tissues after MI by activating NF-κB, which in turn leads to myocardial cell damage and apoptosis.

## Introduction

Myocardial infarction (MI) and subsequent congestive cardiac failure remain the leading cause of mortality and morbidity worldwide (Montecucco et al., 2016). Although early management and advancements in modern medicine have significantly improved prognosis for acute myocardial infarction (AMI), post-MI complications, including recurrent cardiac arrhythmias and reduced cardiac function, remain a leading cause of heart failure (HF) (Velagaleti et al., 2008). Myocardial tissues damaged by ischemia and hypoxia release large quantities of inflammatory cytokines and chemokines, leading to irreversible cardiomyocyte apoptosis (Jhund and McMurray, 2008).

CD47 is a highly glycated, anti-phagocytic molecule expressed on macrophages that is also widely distributed on the surfaces of various cells (Engelbertsen et al., 2019), such as tumor cells (Lo et al., 2015; Zhao et al., 2016; Betancur et al., 2017), red blood cells (Velliquette et al., 2019), and cardiomyocytes (Wang et al., 2016). Recently, multiple studies reported that CD47 is abnormally expressed in various heart diseases (Heidt et al., 2014; Chen et al., 2019; Engelbertsen et al., 2019), resulting in damage to the cardiomyocyte surface, impaired macrophage phagocytotic activity, and reduced clearance of dead cells in the infarct area. Meanwhile, treatment with a CD47 antibody served to augment the clearance of apoptotic bodies in plaque, while decreasing accumulation of apoptotic debris in the necrotic zone and reducing the infarct size (Kojima et al., 2016). Although these studies suggest a new approach for the repair and management of damaged cardiomyocytes, the mechanism underlying CD47 activation during the pathological development of MI remains unclear.

Glycogen synthase kinase 3 beta (GSK-3β), a serine/threonine kinase, contributes to various biological functions (Jope et al., 2007; Hur and Zhou, 2010; Abe et al., 2020), including cell growth, cytoskeleton integrity, cell cycle, and metabolism (Karyo et al., 2010; Shin et al., 2011; Shin et al., 2014). GSK-3β/NF-κB pathway is essential in many processes, including apoptosis, inflammation, and tumorigenesis (Hoeflich et al., 2000; Schwabe and Brenner, 2002). In cardiovascular diseases, GSK-3β is involved in the regulation of cardiomyocyte proliferation (Kerkela et al., 2008), cardiac fibrosis (Lal et al., 2014), myocardial remodeling (Woulfe et al., 2010), and cardiomyocyte apoptosis (Kaga et al., 2006). NF-κB is also a core response regulator of myocardial ischemia and reperfusion injury, the activation of which is involved in the MI post-ventricular remodeling process (Hausenloy et al., 2012). Moreover, NF-κB signaling reportedly regulates CD47 expression in breast cancer cells (Liu et al., 2018). Therefore, in this study, we investigated whether GSK-3β participates in CD47 expression by activating NF-κB signaling after MI. Deciphering the underlying mechanism associated with MI in an effort to identify new treatment targets, has the potential to reduce MI-associated mortality.

## Materials and Methods

### Animals

Eight-week-old male Sprague Dawley rats, weighing 220–250 g, were housed under standard conditions at the experimental animal center of Zhengzhou University (Zhengzhou, Henan, China) at room temperature (25°C), with a humidity of 40 ± 60% and 12-h light-dark cycles. Rats were fed a standard diet and water *ad libitum*. All animal experiments were approved by the Animal Experiments Committee of Zhengzhou University and performed according to the guideline for the Care and Use of Laboratory Animals (NIH Publication, No. 85–23, revised 1996).

### Myocardial Infarction Model

The protocol for the Sprague Dawley rat MI model and drug rationale (SB216763, a potent irreversible and cell-permeable pharmacological inhibitor of GSK-3 which is highly selective for GSK-3β and has no significant influence on the activity of other kinases (Coghlan et al., 2000), 0.6 mg/kg^−1^, administered intravenously 1 h before surgery) were based on our recent report (Wang et al., 2020). Rats were randomly divided into three groups (*n* = 10 each) namely Sham, MI and SB + MI. Following the loss of corneal reflex in all rats, we opened the thoracic cavity and ligated the left anterior descending artery (Sham group: The thread was inserted without ligation after SB216763; the SB + MI group: SB216763 was injected through the tail vein 1 h before ligation). Three heart samples were fixed in 4% formalin for histopathological examination, and seven samples were used for quantitative real-time (qPCR) and western blotting analyses at 7 days.

### Echocardiographic Measurements

Cardiac function was evaluated at seven post-operation by transthoracic echocardiography. The left ventricular ejection fraction (LVEF), left ventricular fractional shortening (LVFS), left ventricular end-diastolic (LVED), and stroke volume (SV) were calculated using M-mode tracing (Vevo 2,100; Visual Sonics, Toronto, Canada).

### Hematoxylin and Eosin (H&E) Staining

Hearts were individually excised and immediately immersed in 4% formaldehyde for 24 h. After fixation and paraffin-embedding, 4-μm-thick sections were cut and stained with H&E for overall morphological evaluation using an optical microscope (BX60; Olympus, Japan). Image acquisition and analysis were performed using ImageJ Launcher (National Institutes of Health, Bethesda, United States). All measurements were performed in a double-blinded manner by two independent researchers.

### Immunohistochemistry

Immunohistochemistry staining of paraffin sections was performed using a microwave-based antigen retrieval method. The heart tissues were fixed in 4% paraformaldehyde and embedded in paraffin. The sections were subsequently cut at 6-μm intervals perpendicular to the long axis of the heart. The primary antibody-rat CD47 (1:200, Abcam, Cambridge, MA, United States) was visualized using Alexa Fluor 550 secondary antibody (1:200, Servicebio, Wuhan, China). The primary antibody-rat CD68 (1:200, Abcam) for macrophages detection was visualized using HRP-labeled secondary antibody (1:200, Servicebio). The nuclei were stained with 4′, 6-diamidino-2-phenylindole dihydrochloride (Invitrogen, New York, United States). CD47/CD68-positive cells per square millimeter were counted under a 20× power field of the microscope in five random areas of LV tissues.

### Apoptosis Assay

For cell apoptosis assays, the FITC-Annexin V apoptosis detection kit (Tianjin Sungene Biotech Co., Ltd.) was used according to the manufacturers’ instructions. Briefly, cells (2 × 10^5^ cells/plate) were incubated in 6-well plates for 48 h. Cells were subsequently collected by mild trypsinization, stained with FITC-Annexin V and propidium iodide on ice for 5 min, and subjected to flow cytometric analysis using analytical flow cytometry (BD FACSymphony™ A5, New Jersey, United States).

### H9c2 Cell Culture

The H9c2 (rat embryonic ventricle) cell line was purchased from the Type Culture Collection of the Chinese Academy of Sciences (Shanghai, China) and cultured in Dulbecco’s modified Eagle medium (DMEM, Corning Inc., Corning, NY, United States of America) supplemented with 10% fetal bovine serum (FBS) and 1% penicillin/streptomycin.

### Isolation and Culture of Primary Rat Neonatal Cardiomyocytes

Rat neonatal cardiomyocytes were harvested according to a previously described method (Wang et al., 2020). Briefly, heart ventricles from newborn rats were used within 24 h of birth. The ventricles were minced and digested with 0.1 mg/ml trypsin (Solarbio, Beijing, China) and 0.1 mg/ml collagenase II (Worthington, Lakewood, NJ, United States). The cell suspensions were plated for 2 h at 37°C to separate rat cardiac fibroblasts from RCMs. The supernatant containing RCMs was collected and inoculated into a 6-well plate, which was treated experimentally 24 h later. The cells were then cultivated in DMEM with 10% FBS and 1% penicillin/streptomycin.

### Transient siRNA Transfection

Rat GSK-3β siRNA and negative control siRNA were obtained from Thermo Fisher Scientific (Invitrogen). H9c2 cells were transiently transfected with 40 pM siRNA using 3.75 µL lipofectamine RNAiMAX (Invitrogen), following the manufacturer’s instructions.

### Cell Hypoxia Model

An anaerobic workstation was maintained to establish the cell hypoxia model (Research Scientific Services, Derwood, MD, United States). RCMs or H9c2 cells were placed in the transfer chamber and exposed to high-purity N_2_. Then, the cells were transferred to the anaerobic working chamber, followed by introduction of mixed gas (10% H_2_ + 10% CO_2_ + 80% N_2_). The cells were subsequently divided into three groups, control (CT); hypoxia model, hypoxia model with SB216763 (10 µM) (Kirby et al., 2012), or SN50 (an NF-κB inhibitor, 18 μM) (Zhao et al., 2013), or CD47 monoclonal antibody (10 μM) (Mohanty et al., 2019) pretreatment for 1 h before hypoxia was performed at various time points (6, 12, and 24 h).

### Quantitative Real-Time PCR

Total RNA was isolated from rat heart tissues using TRIzol (Roche, Germany) and reverse transcribed, following the manufacturer’s protocol (RR047A, Takara Bio, China). Subsequently, qPCR was performed using SYBR Green Master mix (Thermo Fisher Scientific, United States) on the 7,500 Fast Real-Time PCR system (Applied Biosystems; Thermo Fisher Scientific, United States). The thermocycling conditions were as follows: holding at 50°C for 2 min; pre-denaturation at 95°C for 2 min, followed by 15 s at 95°C and then 1 min at 60°C for 40 cycles. The melt curve was at 95°C for 15 s, 1 min at 60°C, 15 s at 95°C, and 15 s at 60°C. The mRNA levels were normalized to *GAPDH* levels. The relative expression was calculated using the change-in-quantification (2^−ΔΔCT^) method. The following primers were used: rat *CD47*: forward, 5′-GCT​TGC​TGG​ATA​CCC​CTG​TT-3′; reverse, 5′-TGC​ATA​GGA​AGT​AGG​CGT​GAG-3′; rat *NF-κB*: forward, 5′-TCT​TGA​GGT​GGC​TGC​TTA​CC-3′; reverse, 5′- CAC​CGT​GTT​CAT​TCC​AGT​GTC-3′; rat *GAPDH*: forward, 5′-TCC​CTC​AAG​ATT​GTC​AGC​AA-3′; reverse, 5′-AGA​TCC​ACA​ACG​GAT​ACA​TT-3′.

### Western Blot Analysis

Samples were solubilized in ice-cold RIPA lysis buffer (Solarbio, Beijing, China) containing a protease inhibitor cocktail (MedChemExpress, Shanghai, China). The protein concentrations were determined using the bicinchoninic acid method (Beyotime, Shanghai, China). Membrane proteins were subjected to SDS-PAGE (Solarbio, Beijing, China) and electrophoretically transferred onto polyvinylidene fluoride membranes (Millipore, United States) and blocked with 5% non-fat dry milk in tris-buffered saline containing 0.1% tween-20 (TBST) for 1 h before incubation with rabbit anti-CD47 antibody (1:1,000, Abcam), anti-NF-κB (p65; 1:1,000, Cell Signaling, Danvers, MA, United States), anti-p-NF-κB (p-p65; 1:1,000, Cell Signaling), anti-Bcl-2 (1:1,000; Proteintech, Rosemont, IL, United States), anti-GSK-3β (1:1,000, Cell Signaling), anti-p- GSK-3β (Ser9; 1:1,000, Cell Signaling), anti-Bax (1:1,000; Proteintech), anti-caspase-3 (1:2,000; Invitrogen), and mouse anti-GAPDH (1:10,000, Proteintech) in 5% non-fat dry milk/TBST overnight. Then, the membranes were incubated with alkaline phosphatase conjugated Affinipure goat anti-rabbit IgG (H + L; 1:10,000, Proteintech) in TBST for 2 h at 37°C. The membranes were visualized using an ECL detection kit (Pierce Biotech, Rockford, IL, United States). The blots were analyzed and quantified using the ImageJ analysis software.

### Co-immunoprecipitation

Whole-cell lysates were obtained through RIPA buffer lysis and incubated with anti-CD47/anti-p65 at 4°C for 12 h, and with protein A/G magnetic beads (Invitrogen) for an additional 2 h. After three washes with cold PBS, the immunocomplexes were analyzed using western blotting.

### Statistical Analysis

Data are presented as mean ± SD. All data were analyzed using SPSS 21.0 (SPSS Inc., Chicago, IL, United States). Statistical comparisons were conducted using unpaired *t* tests between two groups. Statistical differences between multiple groups were compared using ANOVA test followed by Bonferroni post hoc tests. *p* values <0.05 were considered statistically significant. This study followed the principles of blinding and randomization.

## Results

### GSK-3β Inhibition Alleviates Myocardial Dysfunction After Myocardial Infarction

**FIGURE 1:**
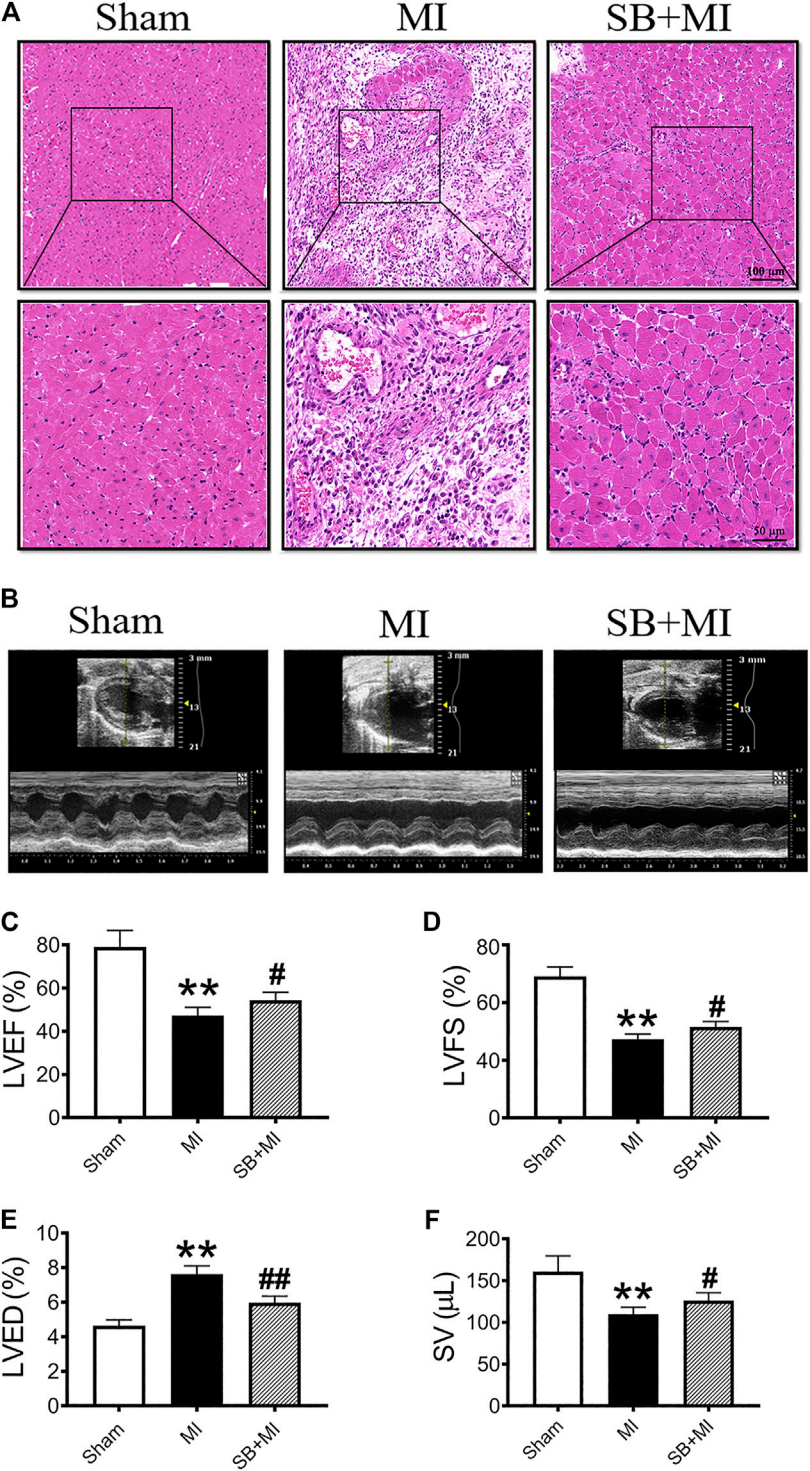
GSK-3β inhibition alleviates cardiac function damage after MI. **(A)**. Representative images of heart sections obtained 7 days after MI and stained with H&E (*n* = 3/group). Scale bars: first row, 100 μm (H&E × 100) and second row, 50 μm (H&E × 200). **(B)** Cardiac function was evaluated using a Vevo 2,100 high-resolution micro-imaging system at 7 days after MI. M-mode echocardiographic imaging obtained 7 days after MI. **(C–F)** Echocardiographic parameter analysis of LVEF **(C)**, LVFS **(D)**, LVED **(E)**, and SV **(F)** (*n* = 7/group). Data are presented as the mean ± SD. ^**^ *p* < 0.01 vs. Sham group; ^*#*^ *p* < 0.05, ^*##*^ *p* < 0.01 vs. MI group.

As shown in Figure 1A, the heart cells of the Sham group were regular in shape and arranged in a tight, orderly configuration. Alternatively, the myocardial structure of the MI group exhibited significant damage, with a loose cell arrangement, broken fibers and blood-filled intercellular spaces. SB216763 pretreatment significantly reduces the myocardial damage caused by the above-mentioned MI.

The number of CD68^+^ cells (a marker of macrophages) in the MI group showed increased compared to those in the Sham group (Supplementary Figure S1A). SB216763 pretreatment significantly reduces the number of CD68^+^ cells (Supplementary Figure S1B).

We then assessed cardiac function after MI using echocardiography (Figure 1B). LVEF (Figure 1C), LVFS (Figure 1D), and SV (Figure 1F) were decreased in the MI group at 7 days post-surgery, while SB216763 treatment significantly reversed these effects. Additionally, the LVED (Figure 1E) was significantly increased in the MI group and decreased by SB216763.

### GSK-3β Participates in CD47 Upregulation in Myocardial Infarction Rats

**FIGURE 2:**
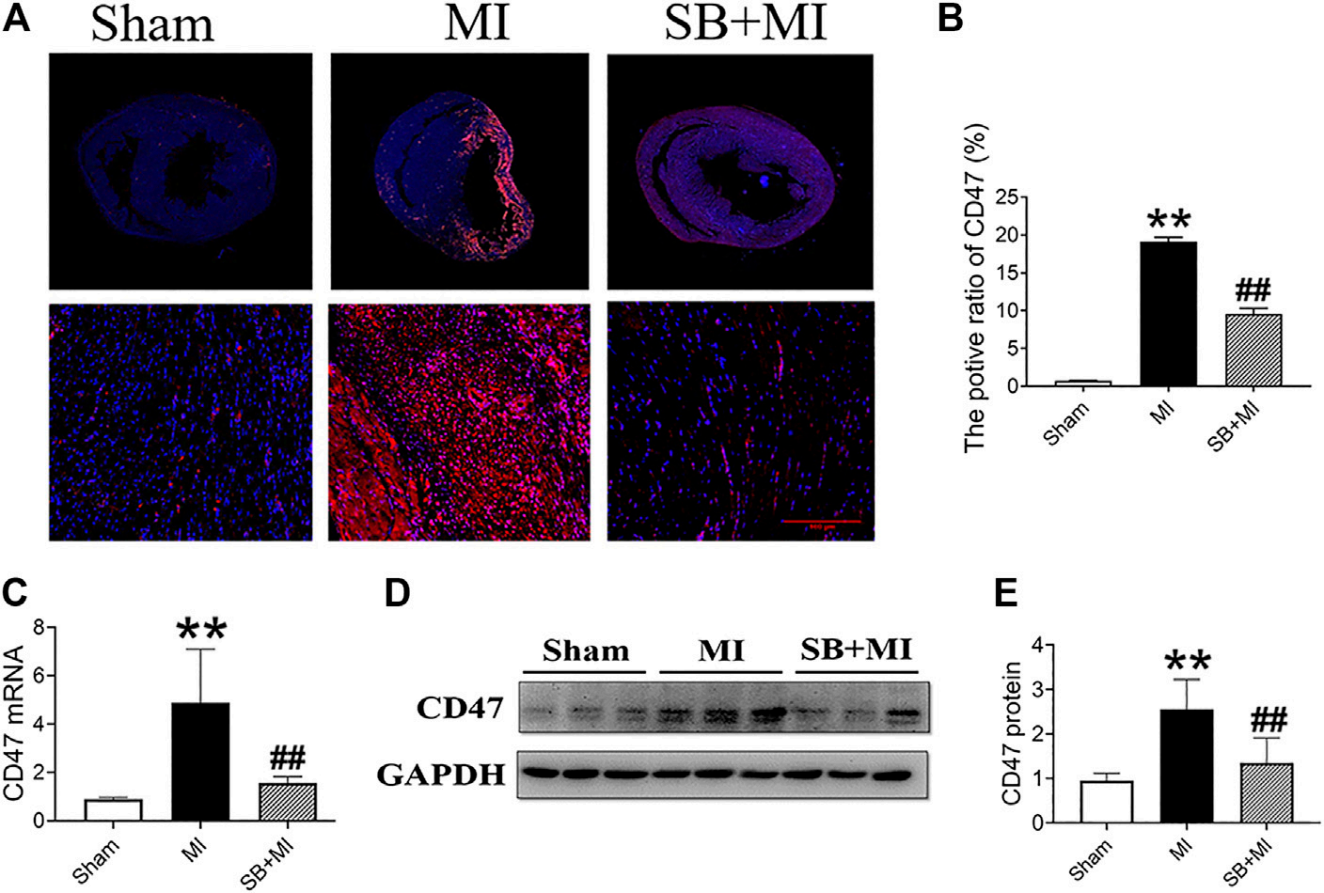
GSK-3β inhibition decreases CD47 expression in rat MI tissues. **(A, B)** Representative immunofluorescence microscopy image of the heart section at 7 days after MI. Immunofluorescence parameter analysis (*n* = 3/group). **(C)** Real-time fluorescence quantitative PCR analysis of CD47 expression in MI tissues of rats at 7 days (*n* = 7/group). **(D, E)** Western blot analysis of CD47 expression in MI tissues of rats at 7 days (*n* = 7/group). Data are presented as the mean ± SD. ^**^ *p* < 0.01 vs. Sham group; ^*#*^ *p* < 0.05, ^*##*^ *p* < 0.01 vs. MI group.

To explore the effect of GSK-3β on CD47 expression in MI tissues, we determined CD47 expression by performing immunofluorescence assays (Figure 2A). The number of CD47-positive cells in the MI group was higher than that in the Sham group, however, decreased after SB216763 pretreatment (Figure 2B). *CD47* mRNA was also significantly upregulated in ischemic tissues compared to the Sham group. SB216763 pretreatment significantly decreased *CD47* mRNA levels compared to those in the MI group (Figure 2C). Similarly, SB216763 pretreatment also decreased the upregulated CD47 protein expression induced by MI (Figures 2D,E).

### GSK-3β Inhibition Suppresses CD47 Upregulation and Apoptosis Under Hypoxic Conditions *in vitro*

**FIGURE 3:**
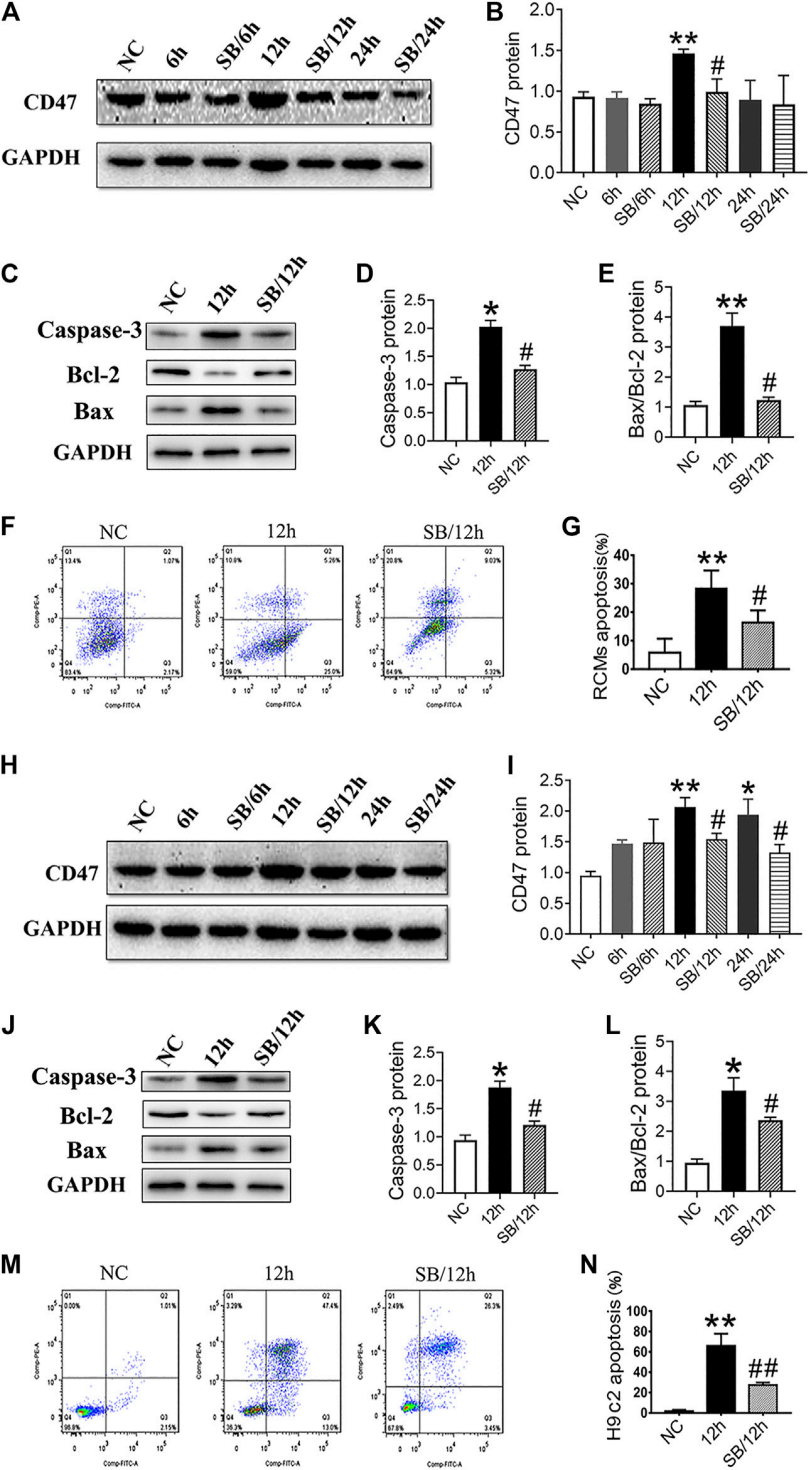
GSK-3β inhibition downregulates CD47 expression and apoptosis in hypoxic cardiomyocytes and H9c2 cells. Western blot analysis of CD47 expression in RCMs **(A, B)** and H9c2 cells **(H, I)**. Western blot analysis of caspase-3, Bcl-2, and Bax expressions in hypoxic RCMs **(C–E)** and H9c2 cells **(J-L)**. Flow cytometry showed a decrease in the apoptosis rate in hypoxic RCMs **(F, G)** and H9c2 cells **(M, N)** (*n* = 3/group). Data are presented as the mean ± SD. ^*^ *p* < 0.05, ^**^ *p* < 0.01 vs. NC; ^*#*^ *p* < 0.05 vs. 12 h, 24 h, ^##^ *p* < 0.01 vs. 12 h.

To further investigate the eﬀects of GSK-3β on CD47 expression, we used RCMs and H9c2 cells to establish an *in vitro* cell hypoxia model*.* Western blotting results showed that hypoxic stimulation increased the expression of CD47 protein, while SB216763 pretreatment decreased its expression in RCMs (Figures 3A,B) and H9c2 cells (Figures 3H,I). In addition, GSK-3β inhibition downregulated apoptosis-related proteins (caspase-3 and Bax/Bcl-2) in RCMs (Figures 3C–E) and H9c2 cells (Figure 3J–L) under hypoxic conditions. Moreover, flow cytometric analysis showed that SB216763 pretreatment reduced the number of apoptotic RCMs (Figures 3F,G) and H9c2 cells (Figures 3M,N).

### GSK-3β Participates in CD47 Upregulation via NF-κB Signaling

Next, we explored whether GSK-3β affects CD47 expression after MI via NF-κB signaling. NF-κB represents a group of structurally related and evolutionarily conserved proteins, with five members in mammals, namely Rel (c-Rel), RelA (p65), RelB, NF-κB1 (p50 and its precursor p105), and NF-κB2 (p52 and its precursor p100), forming homo- or heterodimers that bind the IκB family of proteins in unstimulated cells (Ghosh and Karin, 2002). When cells become stimulated, NF-κB is activated and translocates to the nucleus, through the exposed nuclear localization signal (subunit p65), where it functions to regulate inflammation, cell proliferation, and apoptosis (Silverman and Maniatis, 2001). We, therefore, sought to primarily quantify the expression of the p65 subunit.

**FIGURE 4:**
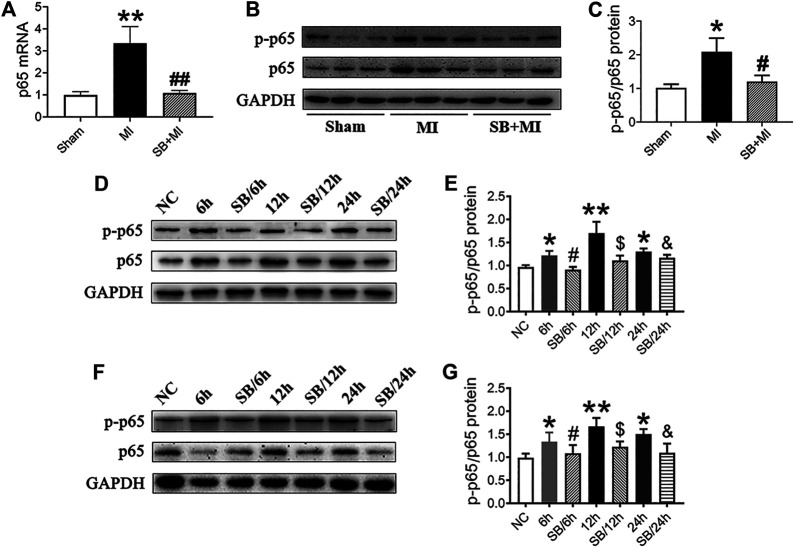
GSK-3β inhibition downregulates NF-κB expression in MI tissues, hypoxic cardiomyocytes and H9c2 cells. **(A)** Real-time fluorescence quantitative PCR analysis of NF-κB expression in MI tissues of rats at 7 days (*n* = 7/group). **(B, C)** Western blot analysis of p-p65/p65 NF-κB in MI tissues of rats at 7 days (*n* = 7/group). Data are presented as the mean ± SD. ^**^ *p* < 0.01, <0.001 vs. Sham group; ^*#*^ *p* < 0.05, ^*##*^ *p* < 0.01 vs. MI group. **(D–G)** Western blot analysis of p-p65/p65NF-κB in RCMs **(D, E)** and H9c2 cells **(F, G)**. Data are presented as the mean ± SD. ^*^ *p <* 0.05, ^**^ *p* < 0.01 vs. NC; ^*#*^ *p* < 0.05 vs. 6 h; ^$^ *p* < 0.05 vs. 12 h; ^&^ *p* < 0.05 vs. 24 h.

As shown in Figure 4A, *NF-κB* mRNA expression increased 7 days after MI, while SB216763 pretreatment reduced this effect. Moreover, ischemic tissues, induced by MI, exhibited an upregulated p-p65/p65 ratio and NF-κB protein levels compared with those in the Sham group. This effect was reversed by SB216763 pretreatment 7 days after MI (Figures 4B,C). Similarly, the level of NF-κB protein increased under hypoxic conditions *in vitro.* Meanwhile, SB216763 pretreatment was found to effectively decrease the upregulated p-p65/p65 ratio and NF-κB protein expression in RCMs (Figures 4D,E) and H9c2 cells (Figures 4F,G).

**FIGURE 5:**
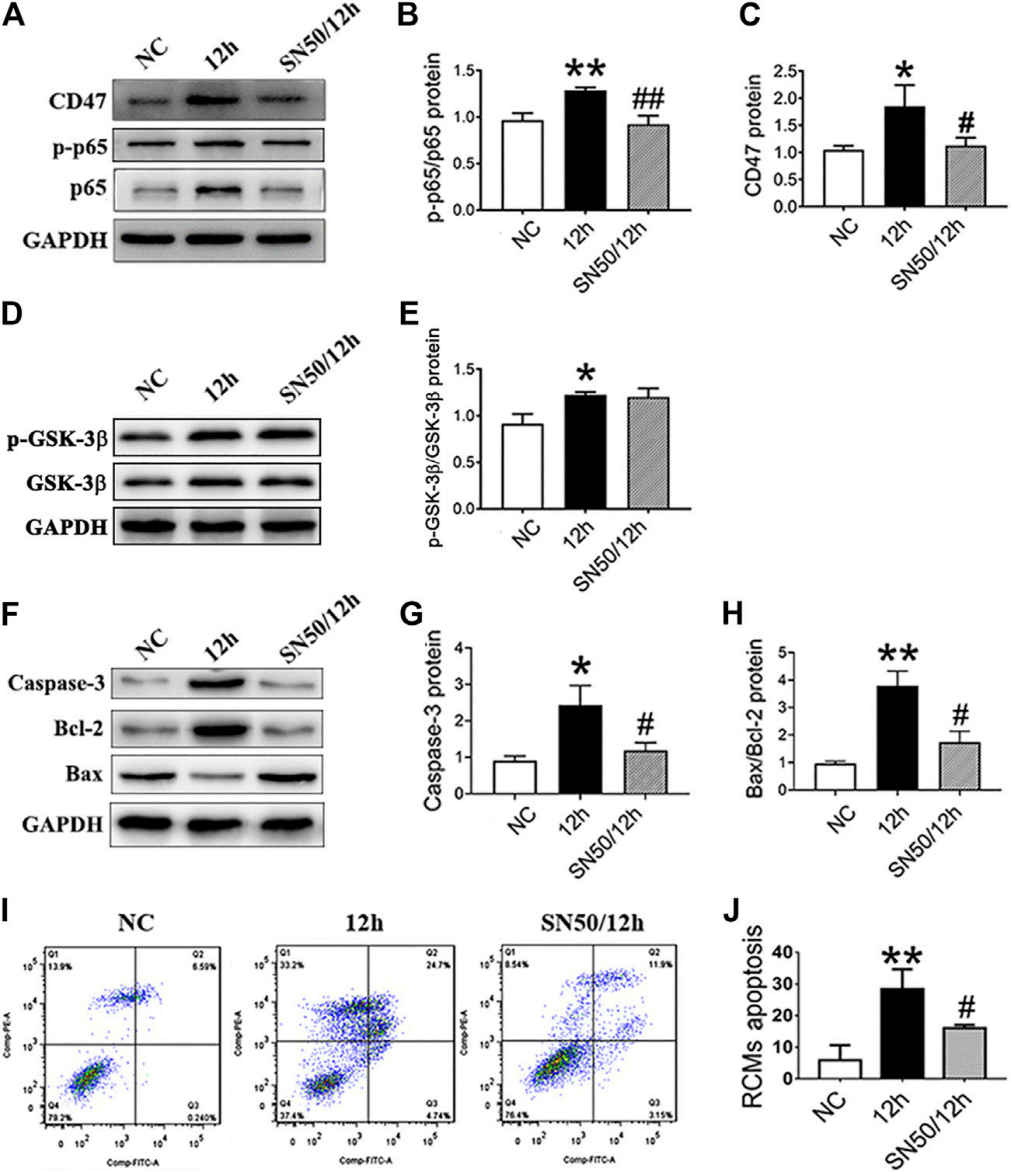
NF-κB inhibition decreases the CD47 expression and apoptosis rate in RCMs. **(A–E)**. Expressions of CD47, p-p65/p65 NF-κB, and p-GSK-3β/GSK-3β in RCMs under hypoxic conditions, as measured using western blot following SN50 pretreatment (18 μM). **(F–H)** Western blot analysis of caspase-3, Bcl-2, and Bax expressions in hypoxic RCMs. **(I, J)** Flow cytometry detected a decrease in the apoptosis rate in hypoxic RCMs following SN50 pretreatment (*n* = 3/group). Data are presented as the mean ± SD. ^*^ *p <* 0.05, ^**^ *p* < 0.01 vs. NC; ^*#*^ *p* < 0.05, ^*##*^ *p* < 0.01 vs. 12 h.

Subsequently, SN50 pretreatment decreased CD47 and NF-κB protein expression (Figures 5A–C), without significantly affecting p-GSK-3β/GSK-3β (Figures 5D,E) under hypoxic conditions. Moreover, SN50 pretreatment reduced apoptosis-related protein expression in RCMs (Figures 5F–H), which was confirmed via flow cytometric analysis (Figures 5I,J).

**FIGURE 6:**
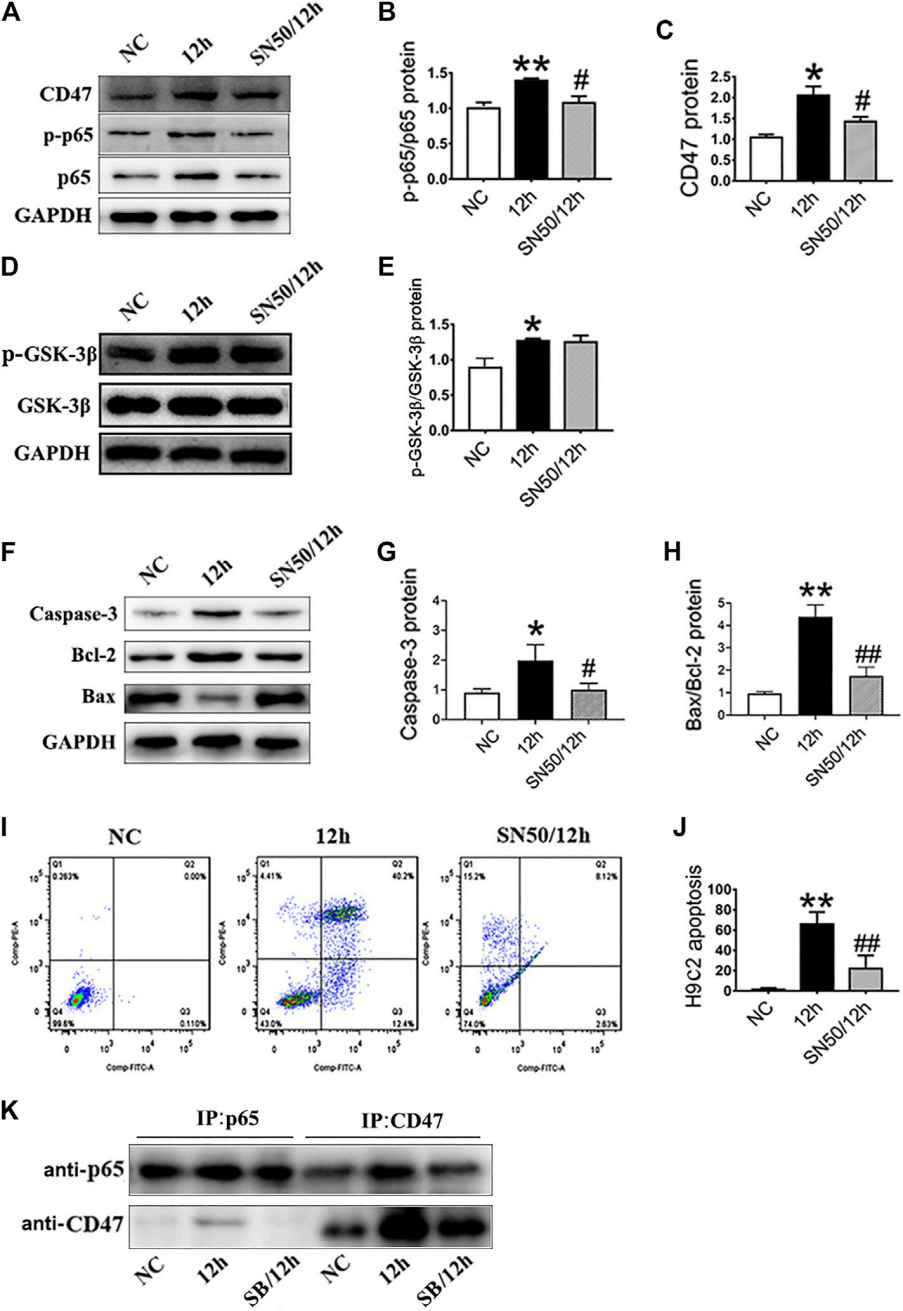
GSK-3β participates in CD47 upregulation and apoptosis via NF-κB signaling in H9c2 cells. **(A–E)**. Expressions of CD47, p-p65/p65 NF-κB, and p-GSK-3β/GSK-3β in H9c2 cells under hypoxic conditions, as measured using western blot following SN50 pretreatment. **(F–H)** Western blot analysis of caspase-3, Bcl-2 and Bax expressions in hypoxic H9c2 cells. **(I, J)** Flow cytometry showed a decrease in the apoptosis rate in hypoxic H9c2 following SN50 pretreatment (*n* = 3/group). **(K)** H9c2 cell lysates were immunoprecipitated with anti-CD47 antibody or anti-p65 antibody, and the resulting immune complexes were analyzed by western blot using various antibodies, as indicated. Data are presented as the mean ± SD. ^*^ *p <* 0.05, ^**^ *p* < 0.01 vs. NC; ^*#*^ *p* < 0.05, ^*##*^ *p* < 0.01 vs. 12 h.

In addition, SN50 elicited similar effects on H9c2 cells in inhibiting CD47 protein upregulation (Figures 6A–C), apoptosis-related proteins (Figures 6F–H), and cells apoptosis (Figures 6I,J) under hypoxic conditions. Meanwhile, SN50 pretreatment did not affect upregulation of hypoxia-induced GSK-3β protein expression (Figures 6D,E). Co-immunoprecipitation further demonstrated the protein interaction between CD47 and p65 NF-κB (Figure 6K). Hence, consistent with our assumption, inhibition of GSK-3β would reduce its interaction with p65.

### Knockdown of GSK-3β Decreases CD47 Upregulation via NF-κB Signaling

**FIGURE 7:**
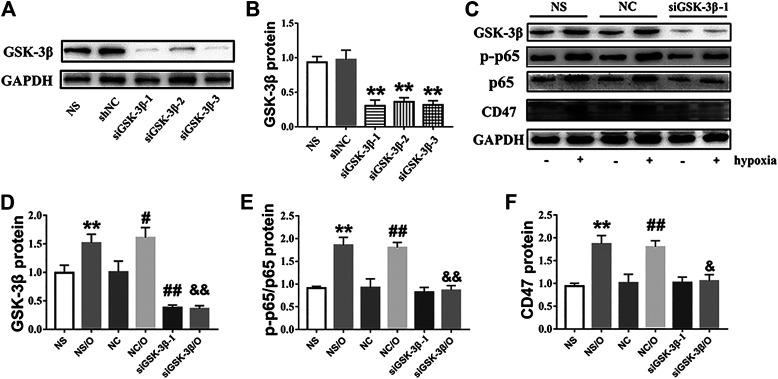
siGSK-3β reduces the expression of CD47 in hypoxia-induced H9c2 cells. **(A, B)**. siGSK-3β-interference efficiency was examined using western blot. ^**^ *p* < 0.01 vs. siNC. **(C)** H9c2 cell lysates of siGSK-3β, siNC, and vehicle control groups after hypoxia induction were analyzed using western blot. **(D–F)** Bar graphs showing fold-changes. “O” stands for hypoxia treatment. ^**^ *p* < 0.01 vs. NS; ^#^ *p* < 0.05, ^##^ *p* < 0.01 vs. NC; ^&^ *p* < 0.05, ^&&^ *p* < 0.01 vs. NC/O.

To examine whether basal GSK-3β depletion exerts protective effects similar to those observed for SB216763, GSK-3β expression was knocked down through transfection with siRNA (siRNAGSK-3β-1, 2, and 3) in H9c2 cells. GSK-3β siRNA knockdown was confirmed by the significant depletion of GSK-3β (Figures 7A,B) and we selected siRNAGSK-3β-1 for further experiments. As shown in Figures 7C–F, knockdown of GSK-3β also decreased NF-κB and CD47 protein upregulation in H9c2 cells.

### Simultaneous Targeting of GSK-3β and CD47 can Significantly Reduce the Apoptosis of Hypoxic Cardiomyocytes

**FIGURE 8:**
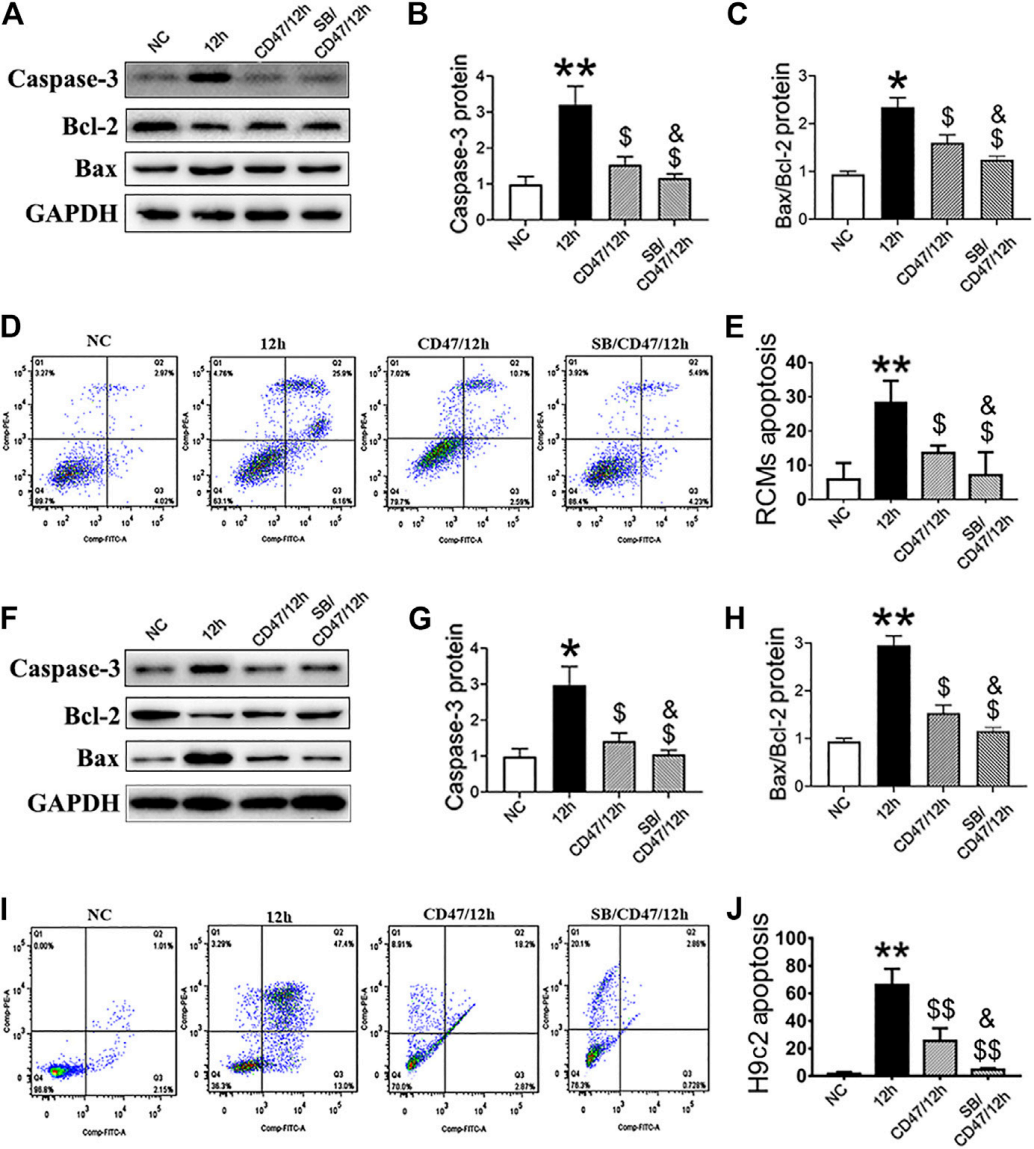
Simultaneous targeting of GSK-3β and CD47 can significantly reduce the apoptosis of hypoxic cardiomyocytes. Western blot analysis of caspase-3, Bcl-2, and Bax expressions in hypoxic RCMs **(A–C)** and H9c2 **(F–H)** cells with inhibited CD47 and GSK-β expression. Flow cytometry showed a decrease in the apoptosis rate in hypoxic RCMs **(D, E)** and H9c2 **(I, J)** cells with inhibited CD47 and GSK-3β expression (*n* = 3/group). Data are presented as the mean ± SD. ^*^ *p* < 0.05, ^**^ *p* < 0.01 vs. NC; ^$^ *p* < 0.05, ^$$^ *p* < 0.01 vs. 12 h; ^&^ *p* < 0.05 vs. CD47/12 h.

RCMs and H9c2 cells were treated with anti-CD47 antibody alone or in combination with SB216763 to evaluate the effect on cell apoptosis. When compared with the NC and 12-h-hypoxia groups, we observed that treatment with anti-CD47 antibody alone sensitized RCMs (Figures 8A–E) and H9c2 cells (Figures 8F–J), while combined anti-CD47 antibody and SB216763 pretreatment exhibited an additive effect on cell apoptosis inhibition.

## Discussion

In this study, we found that targeting GSK-3β can ameliorates acute cardiac injury, improves myocardial dysfunction, and prevents infiltration of inflammatory cells after MI. Moreover, targeting GSK-3β can reduce CD47 expression in MI rats through the NF-κB pathway. In addition, the simultaneous targeting of GSK-3β and CD47 effectively reduced the apoptosis rate of hypoxic cardiomyocytes. Therefore, targeting GSK-3β might represent an attractive therapeutic option for cardiomyocyte repair after MI.

As myocardial cells are highly terminally differentiated and lack proliferation and differentiation ability in a mature state, it is not possible to restore myocardial damage through cell regeneration (Isomi et al., 2019). Although drugs, coronary intervention, and bypass surgery can restore coronary blood flow, myocardial function within the infarct range cannot be restored. It is difficult to reverse the HF process after MI with existing treatments (Braunwald, 2013). Therefore, it is important to identify strategies to overcome the loss of myocardial cells, reduce infarct size, and promote restoration of cardiac function after MI to restrict HF occurrence and development.

CD47 is a ubiquitously expressed transmembrane protein that belongs to the immunoglobulin superfamily and functions as both a receptor for the matricellular protein thrombospondin-1 and a ligand for signal-regulatory protein alpha (SIRPa) (Cheng et al., 2020). Recently, it was found that CD47 is paradoxically upregulated in different cancers (Chao et al., 2011; Willingham et al., 2012). Many studies have reported that tumors evade macrophage phagocytosis and immune surveillance by activating the inhibitory signal via the ligation of SIRPα (which is expressed on phagocytes as a receptor) with CD47 (which is highly expressed on cancer cells as a ligand) (Zhao et al., 2018). Subsequently, CD47 was reported to be highly expressed in cardiovascular diseases, such as atherosclerosis (Kojima et al., 2016), ischemia/reperfusion (I/R) injury (Wang et al., 2016), and HF (Sharifi-Sanjani et al., 2014). Further, continuous upregulation of CD47 has been reported in necrotic cells in carotid and coronary atherosclerosis in both humans and animal models (Chen et al., 2019). Meanwhile, another study reported an increase in CD47 expression in a mouse model of renal I/R injury (El-Rashid et al., 2019). Hence, exploring the regulatory mechanism of CD47 expression might provide a new therapeutic target for heart diseases.

Here, we observed that CD47 expression became upregulated following MI, which is consistent with previously reported data (Chen et al., 2019; El-Rashid et al., 2019). Notably, our results showed that CD47 expression decreased after GSK-3β inhibition. The roles of GSK-3β in cardiac biology are well recognized (Beurel et al., 2015; Lal et al., 2015; Zhou et al., 2016; Sharma et al., 2020). GSK-3β is involved in the development of many cardiovascular diseases via multiple signal transduction pathways, such as Wnt/β-catenin (Guo et al., 2012), TGF-β1-SMAD-3 (Jope et al., 2007; Lal et al., 2014) and apoptosis (Lal et al., 2015; Su et al., 2019). Recently, a study has revealed that targeting GSK-3β by microRNA-99b-3p promotes cardiac fibrosis (Yu et al., 2020). Our present study showed that GSK-3β regulates myocardial fibrotic remodeling in MI via the activation of NLRP3 inflammasome (Wang et al., 2020). Moreover, another study from our lab indicated that inhibition of GSK-3β improved myocardial electrical remodeling by enhancing Kir2.1 expression after MI (Chang et al., 2021). In addition, GSK-3β is critical to cardiac function in high-fat diet-induced obesity (Gupte et al., 2018). Therefore, it is important to better understand the role and regulation of GSK-3β in the pathogenesis of cardiovascular diseases. However, whether, and how, GSK-3β participates in CD47 expression in MI has not yet been elucidated.

NF-κB is a highly conserved nuclear transcription factor that is ubiquitously present in a myriad of cell types. It is involved in specific biological responses that regulate the transcription of target genes and plays an important role in various processes such as immune responses, inflammation, and cell survival (D'Ignazio et al., 2016). A study reported that TNF-NFKB1 signaling can directly regulate CD47 by interacting with a constituent enhancer located within a CD47-associated super-enhancer specific to breast cancer (Liu et al., 2018). Furthermore, the GSK-3β/NF-κB pathway plays important roles in many processes, including I/R injury (Xia et al., 2012), apoptosis, and inflammation (Medunjanin et al., 2016). Our results showed that CD47 and NF-κB protein expression both increased in the infarct area during MI. SB216763 pretreatment decreased the expression of the two proteins. We further observed the same results at the cellular level under hypoxic conditions. To further verify that GSK-3β induced upregulation of CD47 through the NF-κB pathway, we also used inhibitors to limit the NF-κB function and found that inhibiting NF-κB can also reduce CD47 activation and cell apoptosis (Figures 5, 6), without altering the upregulation of hypoxia-induced GSK-3β protein expression (Figures 5D,E, 6D,E), which strongly supports the function of GSK-3β/NF-κB/CD47 axis. Moreover, previous studies have reported that GSK-3β is a regulatory protein upstream of NF-κB (Sathiya Priya et al., 2019; Yang et al., 2020; Yao et al., 2020), which is in line with our findings. We used co-immunoprecipitation to confirm that NF-κB interacts with CD47, and that GSK-3β inhibitor can weaken that interaction. The specific underlying mechanism warrants further investigation. In these experiments, we pharmacologically inhibited GSK-3β and used a siRNA for GSK-3β to validate our findings at the cellular level. Results also indicated that GSK-3β knockdown decreases CD47 upregulation via NF-κB and that CD47 upregulation can be attenuated upon GSK3β inhibition.

Meanwhile, CD47-blockade has only exhibited modest anti-tumor activity, as a monotherapy (Cioffi et al., 2015), since the effect of CD47-blockade is limited (Chen et al., 2017). However, when administered in combination with other target drugs CD47 antibody therapy increases the therapeutic effect (Weiskopf et al., 2013; Martinez-Torres et al., 2015; Liu et al., 2019); hence, we evaluated the effects of anti-CD47 antibody alone or in combination with SB216763 on the apoptosis of hypoxic RCMs and H9c2 cells. Our results showed that the rate of cell apoptosis was reduced following SB216763 treatment alone or anti-CD47 antibody treatment alone, while combination treatment further reduced the rate of apoptosis. These results indicate that targeting GSK-3β (with SB216763 alone or combined with anti-CD47 antibody treatment) can decrease the CD47 expression and apoptosis rate during MI, thereby preserving cardiac function. Recently, studies showed that CD47 inhibition protects against myocardial I/R injury and heart failure (Sharifi-Sanjani et al., 2014; Wang et al., 2016; Zhang et al., 2017; Zuo et al., 2019). Consistent with these observations, our results may provide a novel treatment strategies against ischemic cardiac diseases. We believe that targeting GSK-3β might be a promising target for treating cardiac diseases with more advances in the field and clinical trials.

Certain limitations were noted in this study. First, there are differences in the pathophysiological process of rat and human MI. Although myocardial cell necrosis and scar formation after MI in rats have many of the same characteristics as those in other mammals, it is important to note the limitations of rat models. Second, although primary neonatal cardiomyocytes have been widely used to explore the mechanisms of cardiovascular disorders, differences might exist between neonatal and adult cardiomyocytes. Therefore, we used both RCMs and H9c2 cells to improve the experimental results. Thus, further investigations are necessary to understand the specific roles of CD47 in MI injury.

In conclusion, our results demonstrated that GSK-3β can upregulate CD47 in ischemic tissues and hypoxic myocardial cells by activating NF-κB, resulting in myocardial cell damage and apoptosis (Supplementary Figure S2). Hence, targeting only GSK-3β, or simultaneously targeting GSK-3β and CD47, could significantly reduce hypoxic cardiomyocyte apoptosis. Overall, the findings of this study suggest a new therapeutic target for the repair of myocardial cells following MI to reduce associated mortality.

## Data Availability

The raw data supporting the conclusions of this article will be made available by the authors, without undue reservation.
