# Supplementary figures and images for: Targeting Glycogen Synthase Kinase 3 Beta Regulates CD47 Expression After Myocardial Infarction in Rats via the NF-κB Signaling Pathway
Source: Front Pharmacol. 2021 Jul 19;12:662726. doi: 10.3389/fphar.2021.662726 (PMC8327268; doi:10.3389/fphar.2021.662726)

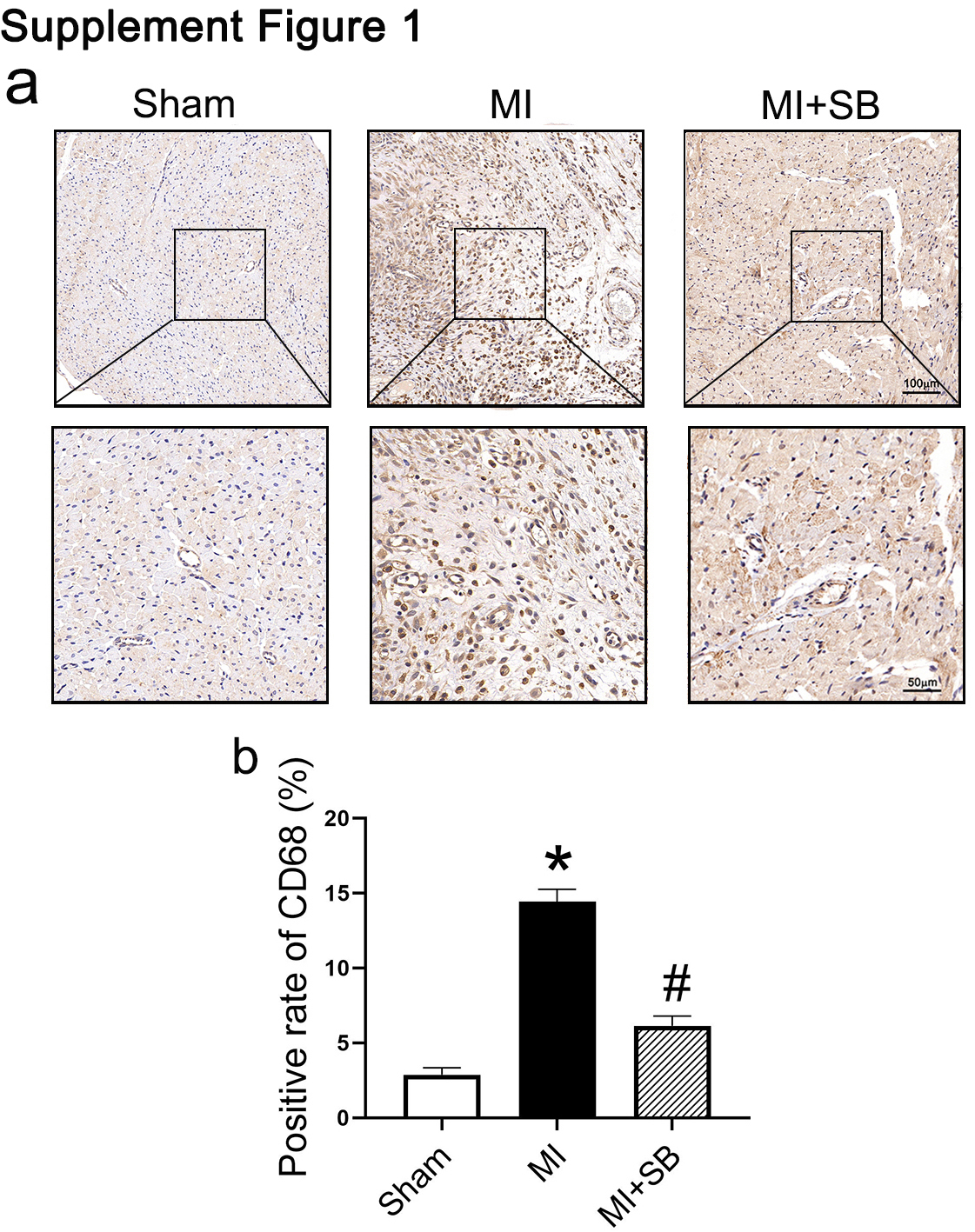

Supplement: Supplementary file 1 [file Image1.jpeg]

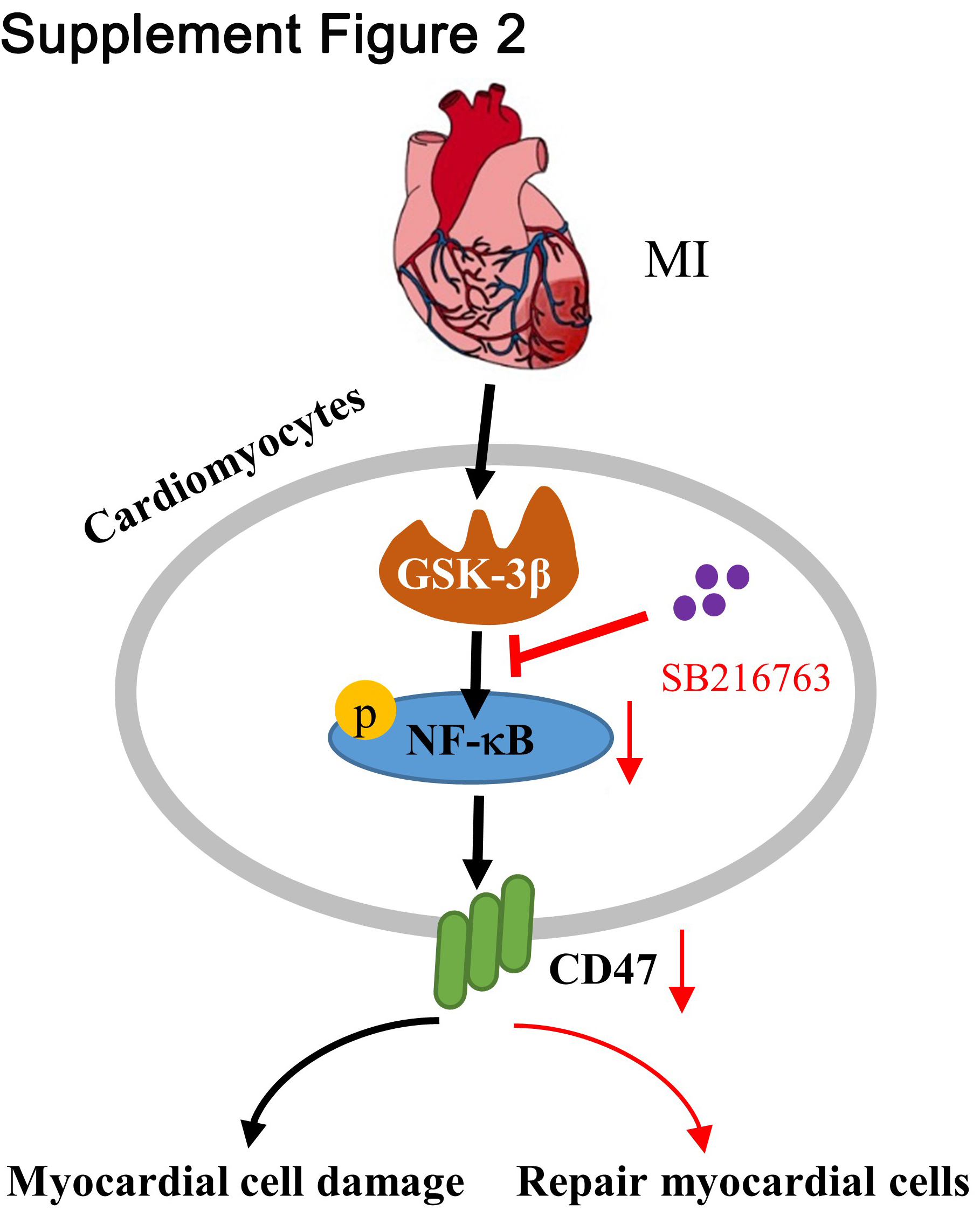

Supplement: Supplementary file 2 [file Image2.jpeg]
